# Effects of footwear with different longitudinal bending stiffness on biomechanical characteristics and muscular mechanics of lower limbs in adolescent runners

**DOI:** 10.3389/fphys.2022.907016

**Published:** 2022-08-19

**Authors:** Hairong Chen, Enze Shao, Dong Sun, Rongrong Xuan, Julien S. Baker, Yaodong Gu

**Affiliations:** ^1^ Faculty of Sports Science, Ningbo University, Ningbo, China; ^2^ The Affiliated Hospital of Medical School, Ningbo University, Ningbo, China; ^3^ Department of Sport Physical Education and Health, Hong Kong Baptist University, Kowloon, Hong Kong SAR, China; ^4^ Center for Health and Exercise Science Research, Hong Kong Baptist University, Kowloon, Hong Kong SAR, China

**Keywords:** running shoes, longitudinal bending stiffness (LBS), adolescents, lower limb biomechanics, muscle mechanics

## Abstract

**Background:** Running shoes with carbon plates have been identified to have positive effects on improving running performance from a biomechanical perspective. However, the specific difference between the effects of carbon plates with different longitudinal bending stiffness (LBS)on biomechanical characteristics and muscular mechanics of lower limbs in adolescent runners remains unclear. This study aimed to identify the difference in biomechanical characteristics and muscular mechanics in lower limbs during running stance phases between wearing shoes with low longitudinal bending stiffness (Llbs) and high longitudinal bending stiffness (Hlbs) carbon plates in adolescent runners.

**Methods:** 10 male adolescent runners with a habit of daily running exercise (age: 13.5 ± 0.6 years; height: 166.3 ± 1.9 cm; bodyweight: 50.8 ± 3.1 kg; foot length: 25.4 ± 0.2 cm) were recruited and asked to conduct two times of tests by wearing shoes with Llbs and Hlbs carbon plates in a randomized order. Paired *t*-test and statistical parametric mapping (SPM) analysis were used to identify the difference in biomechanical characteristics and muscular mechanics in lower limbs during running stance phases.

**Result:** Under the condition of wearing shoes with Hlbs, the time of foot contact significantly increased, whereas the range of motion (ROM) of hip and metatarsophalangeal (MTP) in the sagittal plane significantly reduced as well as the peak moment of ankle joint in the sagittal plane. The activations of vastus medialis, vastus lateralis, flexor digitorum brevis (flex dig brevis), and flexor hallucis longus (flex hall long) significantly increased under the condition of wearing shoes with Hlbs. According to the results of the SPM analysis, the joint angles (hip, ankle, and MTP), the net joint moments (knee, ankle, and MTP), and the muscle forces (gluteus maximus and tibialis anterior) were significant difference during the running stance phase between conditions of wearing shoes with Hlbs and Llbs.

**Conclusion:** Running shoes with Llb carbon plates are appropriate for adolescent runners due to the advantages of biomechanical characteristics and muscular mechanics.

## Introduction

Running, which is a flexible and practical exercise with freedom in time and place, has gradually become one of the most popular leisure sports with an accumulating amount of youthful participants due to its benefit for health (Baranowski et al., 1997). Within the development of running shoes, the role of carbon plates embedded in the button of running shoes are becoming increasingly important. Moreover, because of the potential in providing positive effects on performance during the ground contact phases of walking, running, and jumping, the longitudinal bending stiffness (LBS) of the carbon plates in shoes has attracted considerable attention from biomechanics researchers in recent decades (Flores et al., 2019).

Previous studies have demonstrated that a larger LBS of shoes could improve the performance of vertical jumping and sprinting as well as optimize the energy efficiency of running (Stefanyshyn and Nigg, 2000; Stefanyshyn and Fusco, 2004; Roy and Stefanyshyn, 2006). Since many previous studies found that the increased LBS could reduce the dorsiflexion of the metatarsophalangeal joint (MTP) as well as the ground reaction forces (GRFs) for propulsion, the mechanism of running energy efficiency optimization might be that the reduction of oxygen consumption during running-induced by the biomechanical advantages from the increased LBS (Madden et al., 2016; Hoogkamer et al., 2019; Jiang 2020). For example, Roy and Stefanyshyn (2006) reported that the increased LBS resulted in the increased peak ankle joint moment. Ortega’s study published in 2021 claimed that the MTP and ankle joint mechanics were primarily affected by LBS, which had little effect on knee and hip joint mechanics (Ortega et al., 2021). At the same time, as the LBS increased, in the sagittal plane, the center of GRFs moved forward and the moment arm of GRFs of lower limb joints increased (Willwacher et al., 2014; Madden et al., 2016; Moore et al., 2016; Flores et al., 2017). A systematic review and meta-analysis conducted by Rodrigo-Carranza’s team in 2021 identified that, in comparison to normal shoes, it would take more force generated by muscle to bent shoes with a high longitudinal bending stiffness (Hlbs). Besides, this review also claimed that individuals tend to increase their plantar ankle flexion moments and muscle contractions to overcome mechanical disadvantage caused by the prolonged foot contact and propulsive phases induced by the LBS increase (Rodrigo-Carranza et al., 2021). A study conducted by Cigoja’s team in 2020 also found that, when compared to the participants who wore normal shoes in the control group, the participants who wore Hlbs shoes in the experimental group had a lower shortening rate of calf triceps tendons. They inferred that to generate equivalent muscular force under a slower shortening rate of muscle fiber, fewer motor units were recruited. By this mechanism, the metabolic expenditure during running was cut since the muscular power output had become more economical (Cigoja et al., 2020). In addition, other kinematic and kinetic data have been shown to correlate with energy expenditure while running (Hoogkamer et al., 2018). According to the present evidence, the higher LBS of the shoe could indicate a better running economy. A study by Roy and Stefanyshyn (2006) examined the running economy (RE) when wearing shoes of three different LBS conditions and found that being compared to wearing normal shoes, the RE of runners would increase by 0.8 percent when wearing shoes with Llbs carbon plates. In contrast, the increase of RE when wearing shoes with Hlbs carbon plates seemed not statistically significant when being compared to that of wearing normal shoes.

Previous studies have verified that different mechanical characteristics of the muscle-tendon complex in lower limbs could affect the performance, energy expenditure, and biomechanical characteristics of running (Beltran, 2021). For example, generally speaking, males have stiffer muscle-tendon complexes in lower limbs than females (Kong and De Heer, 2008). Concerning the effect of such physiological differences on lower limb biomechanics, some previous studies have revealed the gender difference in lower limb biomechanical characteristics in runners wearing shoes with different LBS (Micheli et al., 2013). However, most previous studies focused on the biomechanical characteristics and muscular mechanics of adult runners or physiological parameters of adolescent runners, for instance, in 1989, Nudel’s team found that the factors affecting the running performance of adolescents were related to blood lactic acid level, bone maturity, and body coordination under the influence of maximal oxygen uptake (Nudel et al., 1989). In 2021, Xu’s team found that the body of adolescents is undergoing growth and maturing such as the longitudinal growth that occurs in the articular cartilage and epiphysis and axial isotope growth in the thickening and broadening of long bones (Xu et al., 2021), few studies focus on the biomechanical characteristics and muscular mechanics of lower limbs in adolescent runners.

Unsimilar to adult runners whose musculoskeletal systems are more balance within bones, muscles, and soft tissues and hamstrings and quadriceps usually contain more muscle fibers, adolescents, whose bone expansion would possibly outpace the muscle synthesis and tendon lengthening, are easy to get a “mismatch” in their musculoskeletal systems because of their rapid body growth (Kong and De Heer, 2008). Moreover, adolescent runners have lower muscle strength than adults but often have more joint mobilities and tendon flexibilities (Micheli et al., 2013). Therefore, research focusing on the biomechanical characteristics and muscular mechanics of lower limbs in adolescent runners would provide a significant contribution to the field.

This study aimed to identify the difference in biomechanical characteristics and muscular mechanics in lower limbs during running stance phases between wearing shoes with Llbs and Hlbs carbon plates in adolescent runners based on the following hypotheses. First, the range of motion (ROM) of the MTP will reduce when wearing shoes with Hlbs whereas the peak moment of the ankle will increase. Second, during the running stance phases, the peak muscle force generated when wearing shoes with Llbs carbon plates will be smaller than that generated when wearing shoes with Hlbs carbon plates. Last, during the running stance phases, the impulsion loaded on lower limbs when wearing shoes with Hlbs will be larger than that loaded when wearing shoes with Llbs.

## Materials and methods

### Participants

10 adolescent runners, who have a habit of running exercise (more than 16 km of running exercise in 1 week, more than 70 km of running training in the last month, and have been receiving any form of aerobic training over the previous 2 years) were recruited (Pereles et al., 2012). The volunteers, who had no lower limbs musculoskeletal injury history during the past 2 months and with shoe size at 41 in European size, right leg as the dominant leg, and heel-grounding running, were screened out as the participants in this trial. The mean age of the eligible participants was 13.5 ± 0.6 years old, the mean body height was 166.3 ± 1.9 cm, the mean body weight was 50.8 ± 3.1 kg, and the mean foot length was 25.4 ± 0.2 cm. All participants obtained and signed written consent forms approved by the Institutional Review Board before the test.

### Experimental procedures

**FIGURE 1:**
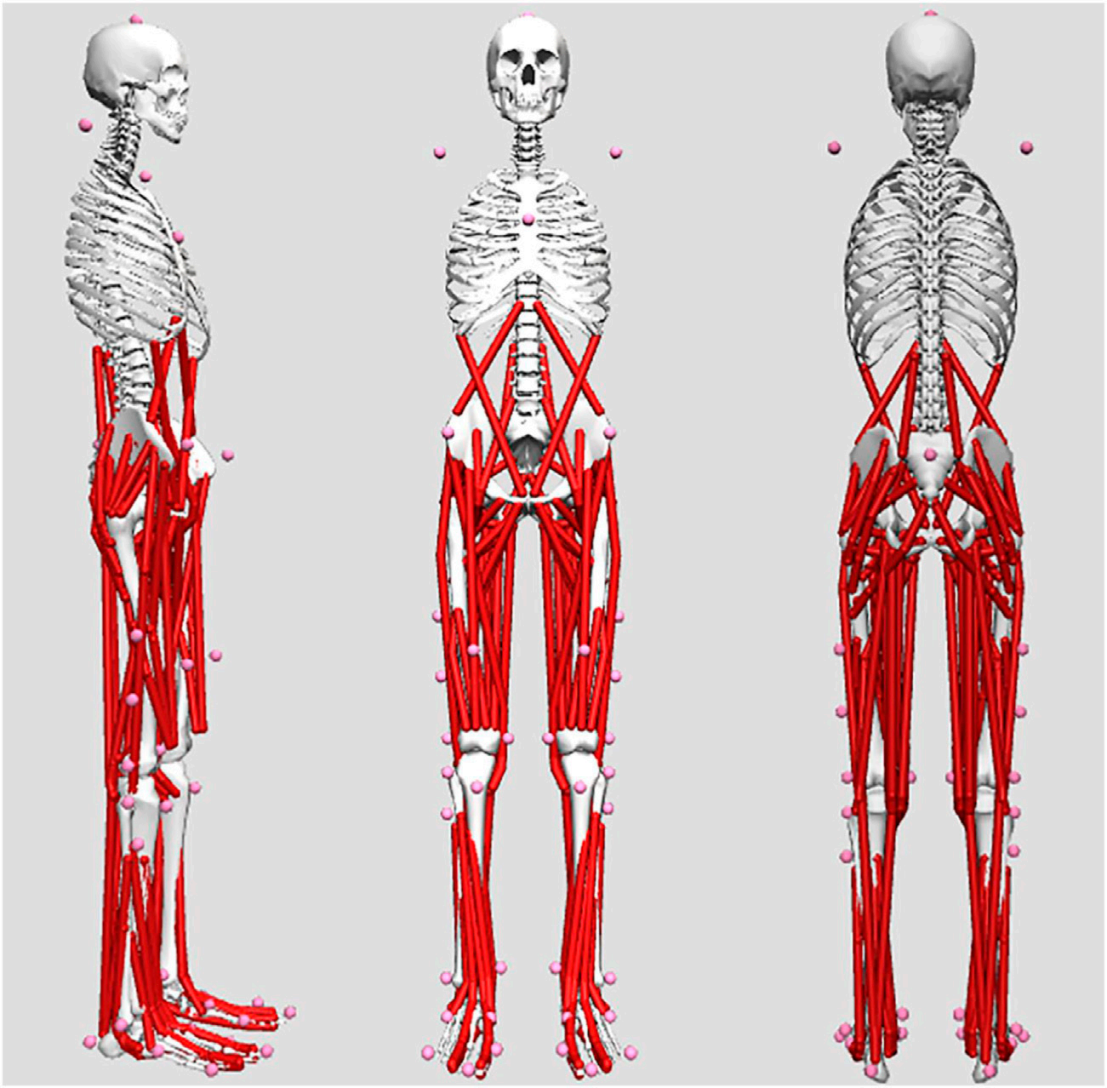
Illustration of the placements of the marker on three different sides.

The information on running shoes that were used in the test is shown in Table 1. The specific LSB values were 5.0 Nm/rad (Llbs shoes) and 8.6 Nm/rad (Hlbs shoes); the difference in LBS between Hlbs shoes and Llbs shoes was that a 1 mm carbon plate is added to Llbs shoes, while a 1.5 mm carbon plate is added to Hlbs shoes; the rest of the shoe was the same. Shoe longitudinal bending stiffness was tested using a 3-point bending protocol (Roy and Stefanyshyn, 2006). All tests were conducted in the laboratory of biomechanics. The kinematic data were collected using a VICON MX motion analysis system (Oxford Metrics Ltd., Oxford, United Kingdom), which consisted of eight cameras and sampled at a rate of 200 Hz. The dynamics data were collected at a sampling rate of 1,000 Hz using a 600 mm × 400 mm force platform (AMTI, Watertown, MA, United States). Synchronization of the kinematic and kinetic data was performed. Reflective markers were placed following the gait 2392 marker set (Kim et al., 2018). Each participant was fitted with thirty-nine (12.5 mm in diameter) reflective markers. Referring to previous research methods and good wrapping of shoes, foot markers were placed on the shoe (Cigoja et al., 2019). Figure 1 outlines the placement of each marker.

**TABLE 1:** List of experimental running shoes.

Llbs shoes (5.0 Nm/rad, 1.0 mm carbon plate)	Hlbs shoes (8.6 Nm/rad, 1.5 mm carbon plate)
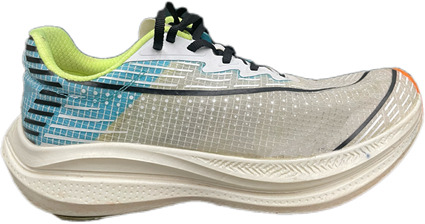	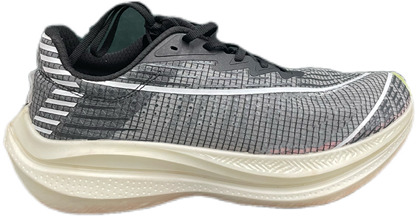

### Procedure

Each participant was required to complete a warm-up session including one set of 10-min running on a treadmill at 8 km/h and one series of lower-body stretches (Zhou et al., 2021). All participants were allowed three trials to familiarize themselves with the test maneuvers before the formal test. In the first step of the formal test, each participant was asked to stand on a force platform to collect static coordinates by standing parallel to the *Y*-axis of the force platform with arms crossed over shoulders and eyes looking forward until the full static coordinates were captured. Then, each participant performed running tasks along 10 m sidewalks at a speed of 3.3 m/s with 1minunte interval rest for kinematic and kinetic data collection (Isherwood et al., 2021). After obtaining 5 times trials with eligible data on the dominant leg, in which the running speed of the participant was less than 5% variance and within 5% of the predefined running speed. The definition of the initial contact was the moment when the ground reaction force exceeded 10 N (Jiang et al., 2021).

### Data process and analysis

The kinematic data and ground reaction force were identified and collected by the Vicon Nexus 1.8.6 software and exported as c3d format files. MATLAB R2016b (The MathWorks, Natick, MA, United States) was used for coordinate system transformation, low-pass filtering, data extraction, and format conversion of kinematic data and ground reaction force data. The process of data analysis was as followed: 1) Convert the coordinate system of the kinematic data and ground reaction force data to the coordinate system used in subsequent simulations. The forward direction of the human body was defined as the positive direction of the *X*-axis, and the upward direction perpendicular to the ground was the *Y*-axis. The positive direction and the right direction of the human body were the positive directions of the *Z*-axis. 2) Biomechanical data of marker trajectories and ground reaction forces filtered with 6 and 30 Hz fourth-order zero-phase lag Butterworth low-pass filters. 3) Extract the kinematic and ground reaction force data of running stance phases and convert the data format to track (Marker track) and mot. (Force plate data) format required by OpenSim simulation software. OpenSim (Stanford University, Stanford University, CA, United States) was used to process and calculate biomechanical parameters in our research. The musculoskeletal model from OpenSim (gait 2392) was used, which had ten rigid bodies, 23 degrees of freedom, and 92 tendon actuators (Delp et al., 1990; Delp et al., 2007). In the following steps, lower limb joint angles, joint moments in the sagittal plane, and muscle force were outputted.

Step 1: Import the statics model into the OpenSim 4.2 software. Then use the scale tool to obtain the anthropometric model of each participant. Identify the muscle starting and ending points, and ensure the moment arms were consistent with the length of the participants’ limb (Delp et al., 2007). Step 2: Use the inverse kinematics (IK) tool in the OpenSim 4.2 software to calculate the joint ROM during the running stance phases and create a motion file (mot). Simultaneously, use the inverse dynamics tool to import the running marker files and external force files into OpenSim and then calculate the joint moments of all the participants. Step 3: Using a static optimization tool with the kinematic data calculated and motion file, calculate the muscle force (Delp et al., 1990). Joint moments and muscle forces were normalized to body weight.

### Statistical analysis

The statistical calculations were carried out using SPSS version 25.0 software (IBM, Armonk, NY, United States). Paired *t*-tests were used to identify the difference in kinematic and kinetic parameters in lower limbs during running stance phases between wearing shoes with Llbs and Hlbs carbon plates. A Holm-Bonferroni correction was implemented to account for multiple *t*-tests. The criterion of statistical signification was set at 0.05.

The kinematic and kinetic data characteristics were one-dimensional and time-varying (Jiang et al., 2021). Paired *t*-test was applied to compare mean joint angles, moments, and muscle forces during the running stance phases by using one-dimensional statistical parametric mapping (SPM1d), which relied on random vector field theory to account for data variability (Pataky et al., 2017). The statistical analyses were conducted in MATLAB R2016b (The MathWorks, Natick, MA, United States) with the criterion of statistical signification was 0.05. A Holm-Bonferroni correction was implemented to account for multiple *t*-tests.

## Results

The contact time increased under the Hlbs condition with a statistical significance (*p* = 0.046); the ROM of the sagittal plane hip angle and MTP joint angle reduced under the Hlbs condition with a statistical significance (*p* = 0.004 and *p* = 0.026); the peak moment of the sagittal plane ankle moment reduced under the Hlbs condition with a statistical significance (*p* = 0.043). (Table 2).

**TABLE 2:** Average contact time, ROM, and peak moment of lower limb joints during stance phase in the Llbs and the Hlbs shoes (mean ± SD).

Variable	Llbs	Hlbs	Within-subject change (%)	*p*-value
Contact time (s)	0.27 ± 0.03	0.30 ± 0.03	15.08 ± 12.79	**0.046**
Hip sagittal ROM (°)	50.01 ± 5.70	38.80 ± 2.97	−21.59 ± 10.32	**0.004**
Knee sagittal ROM (°)	33.76 ± 7.68	36.15 ± 6.27	8.53 ± 10.14	0.999
Ankle sagittal ROM (°)	46.73 ± 3.14	45.48 ± 2.76	−2.41 ± 6.93	0.999
MTP sagittal ROM (°)	17.27 ± 3.24	13.28 ± 2.38	−22.39 ± 12.18	**0.026**
Peak hip moment (N.m/kg)	2.50 ± 0.34	2.36 ± 0.40	−3.44 ± 22.21	0.999
Peak knee moment (N.m/kg)	3.53 ± 0.53	3.30 ± 0.30	−5.71 ± 6.77	0.621
Peak ankle moment (N.m/kg)	3.52 ± 0.39	3.14 ± 0.46	−10.91 ± 8.76	**0.043**
Peak MTP moment (N.m/kg)	0.47 ± 0.07	0.45 ± 0.03	−3.59 ± 12.68	0.999

Note: Average within-subject changes are reported as a percentage difference (Hlbs-Llbs)/Llbs×100%; *p*-value in bold when significant (*p* < 0.05), statistical difference between shoe conditions. Bold fonts represent statistical differences, *p* < 0.05.

**FIGURE 2:**
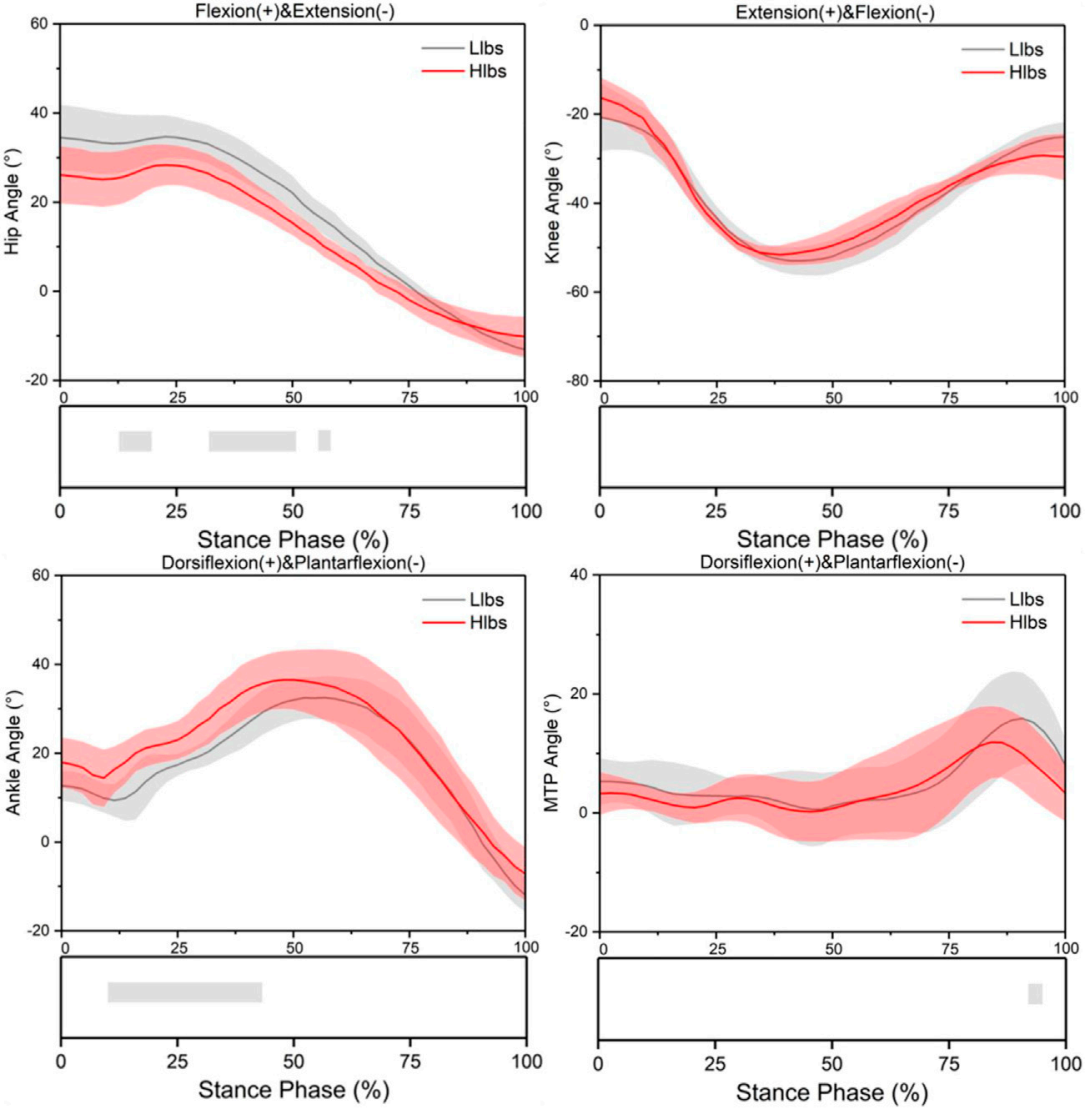
Lower limb joint angle waveforms of mean and standard deviation over the stance phase of two shoe conditions. Significant differences (*p* < 0.05) are highlighted (grey horizontal bars at the bottom of the figure) during corresponding periods from SPM1d analyses.

**FIGURE 3:**
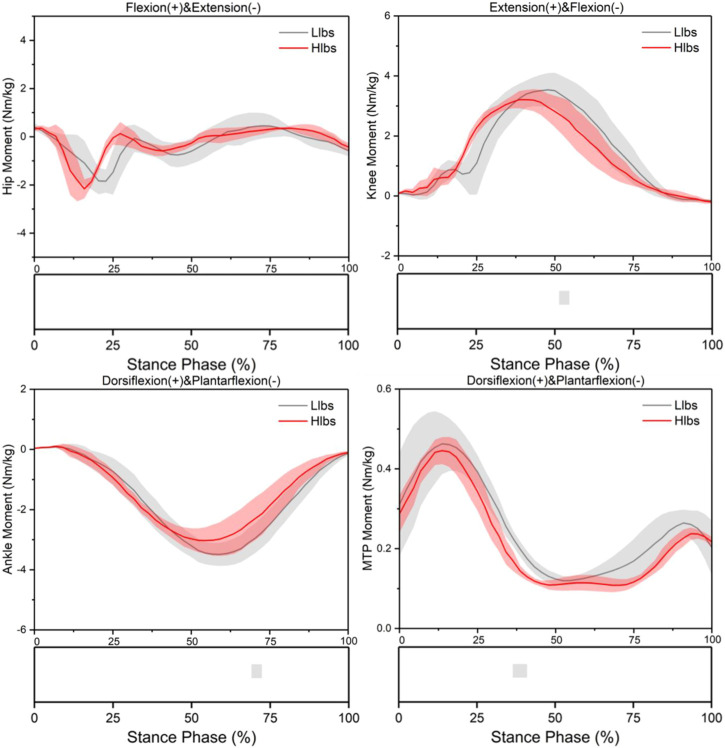
Lower limb joint moment waveforms of mean and standard deviation over the stance phase of two shoe conditions. Significant differences (*p* < 0.05) are highlighted (grey horizontal bars at the bottom of the figure) during corresponding periods from SPM1d analyses.

The flexion angle of the hip in the sagittal plane under the Hlbs condition reduced significantly during the 13.3%–20%, 32.2%–51.1%, and 54.4%–56.7% of the stance phase (*p* < 0.001) (Figure 2). The extension moment of the knee in the sagittal plane under the Hlbs condition reduced significantly during the 51.1%–53.3% of the stance phase (*p* < 0.001) (Figure 3). The dorsiflexion angle of the ankle in the sagittal plane under the Hlbs condition increased significantly during the 10.0%–46.7% of the stance phase (*p* < 0.001); the plantarflexion moment of the ankle in the sagittal plane under the Hlbs condition increased significantly during the 71.1%–73.3% of the stance phase (*p* = 0.001) (Figures 2,3). The dorsiflexion angle of the MTP joint in the sagittal plane under the Hlbs condition reduced significantly during the 93.3% and 95.6% of the stance phase (*p* = 0.001); the dorsiflexion moment of the MTP joint in the sagittal plane under the Hlbs condition reduced significantly during 35.6%–38.9% of the stance phase (*p* < 0.001) (Figures 2,3).

**FIGURE 4:**
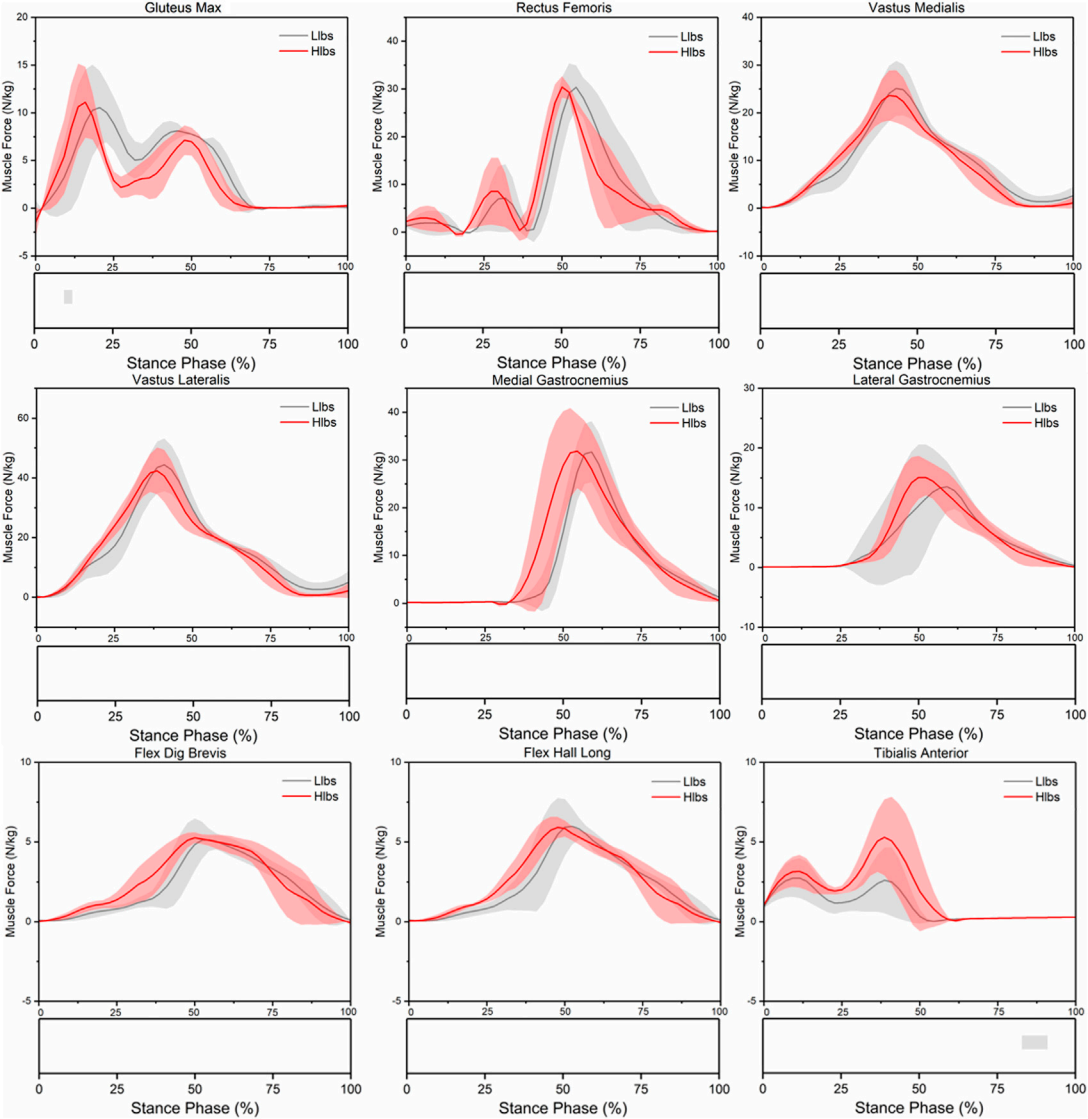
Lower limb muscle force waveforms of mean and standard deviation over the stance phase of two shoe conditions. Significant differences (*p* < 0.05) are highlighted (grey horizontal bars at the bottom of the figure) during corresponding periods from SPM1d analyses.

There was no significant difference in peak muscle force during stance phase in the Llbs and the Hlbs shoes (Table 3). The impulse of vastus medialis, vastus lateralis, flexor digitorum brevis (flex dig brevis)), and flexor hallucis longus (flex hall long) under the Hlbs condition increased significantly (*p* = 0.013, *p* = 0.018, *p* = 0.001, and *p* = 0.016) (Table 4). The muscle force of the gluteus maximus2 (gluteus max2) under the Hlbs condition increased significantly during the 21.1% and 25.6% of the stance phase (*p* < 0.001) (Figure 4); the muscle force of the tibialis anterior under the Hlbs condition increased significantly during the 82.2%–93.3% of the stance phase (*p* < 0.001) (Figure 4).

**TABLE 3:** Peak muscle force during stance phase in the Llbs and the Hlbs shoes (mean ± SD).

Muscle name	Llbs	Hlbs	Within-subject Change (%)	*p*-Value
Gluteus max2 (N/kg)	14.03 ± 1.49	13.88 ± 3.23	−1.51 ± 18.43	0.999
Rectus femoris (N/kg)	31.71 ± 2.80	29.64 ± 4.62	−7.03 ± 9.76	0.702
Vastus medialis (N/kg)	28.11 ± 5.80	27.28 ± 6.22	−3.26 ± 4.05	0.999
Vastus lateralis (N/kg)	47.32 ± 7.70	46.26 ± 8.09	−2.37 ± 3.92	0.999
Medial gastrocnemius (N/kg)	36.65 ± 4.05	35.30 ± 6.68	−3.89 ± 12.83	0.999
Lateral gastrocnemius (N/kg)	16.61 ± 3.46	17.10 ± 1.96	5.35 ± 15.66	0.999
Flex dig brevis (N/kg)	5.55 ± 0.71	5.42 ± 0.27	−0.72 ± 13.70	0.999
Flex hall long (N/kg)	7.09 ± 0.68	6.46 ± 0.45	−8.40 ± 8.21	0.324
Tibialis anterior (N/kg)	4.57 ± 1.69	6.04 ± 2.08	67.51 ± 113.27	0.999

Note: Average within-subject changes are reported as a percentage difference (Hlbs-Llbs)/Llbs×100%; *p*-value in bold when significant (*p* < 0.05), statistical difference between shoe conditions. Gluteus max2, gluteus maximus2; Flex dig brevis, flexor digitorum brevis; Flex hall long, flexor hallucis longus.

**TABLE 4:** Impulse during stance phase in the Llbs and the Hlbs shoes (mean ± SD).

Muscle name	Llbs	Hlbs	Within-subject change (%)	*p*-value
Gluteus max2 (Ns/kg)	1.04 ± 0.10	0.89 ± 0.16	−14.169 ± 14.31	0.297
Rectus femoris (Ns/kg)	1.88 ± 0.55	2.13 ± 0.40	17.76 ± 22.59	0.999
Vastus medialis (Ns/kg)	2.35 ± 0.24	2.60 ± 0.20	10.74 ± 7.03	**0.013**
Vastus lateralis (Ns/kg)	4.10 ± 0.38	4.60 ± 0.25	12.80 ± 8.64	**0.018**
Medial gastrocnemius (Ns/kg)	1.99 ± 0.22	2.76 ± 0.83	36.71 ± 30.97	0.147
Lateral gastrocnemius (Ns/kg)	1.07 ± 0.48	1.36 ± 0.28	42.00 ± 42.59	0.177
Flex dig brevis (Ns/kg)	0.57 ± 0.07	0.72 ± 0.04	27.98 ± 16.83	**0.001**
Flex hall long (Ns/kg)	0.61 ± 0.09	0.74 ± 0.04	22.40 ± 16.26	**0.016**
Tibialis anterior (Ns/kg)	0.29 ± 0.11	0.50 ± 0.19	112.79 ± 137.89	0.999

Note: Average within-subject changes are reported as a percentage difference (Hlbs-Llbs)/Llbs×100%; *p*-value in bold when significant (*p* < 0.05), statistical difference between shoe conditions. Gluteus max2, gluteus maximus2; Flex dig brevis, flexor digitorum brevis; Flex hall long, flexor hallucis longus. Bold fonts represent statistical differences.

## Discussion

The objective of this study was to identify the difference in biomechanical characteristics and muscular mechanics in lower limbs during running stance phases between wearing shoes with Llbs and Hlbs carbon plates in adolescent runners. It is found that the ROM of the hip and MTP, the peak moment of the ankle reduced when wearing shoes with the Hlbs, and the impulse of vastus medialis, vastus lateralis, flex dig brevis, and flex hall long increased when wearing shoes with the Hlbs. When wearing shoes with the Hlbs, the flexion angle of the hip reduced during the 13.3%–20%, 32.2%–51.1%, and 54.4%–56.7% of the stance phase, and the dorsiflexion angle of the ankle increased during the 10.0%–46.7% of the stance phase, and the muscle force of tibialis anterior increased during the 82.2%–93.3% of the stance phase.

Considering the musculoskeletal development of adolescents, in this trial, the running shoes for tests with different LBS were chosen according to the running shoes for adult runners. The existing running shoes on the market such as Nike Zoom Streak 6 and Adidas adizero Adios BOOST 2 have an LBS from 9.4 Nm/rad to 7.0 Nm/rad respectively (Hoogkamer et al., 2019). Day and Hahn defined 10 Nm/rad as the threshold of stiffness in the allocation of experimental groups with different LBS as variate in their trial that explored the optimal LBS to improve running economy (Day and Hahn, 2020). According to this information, 8.6 Nm/rad was chosen as the threshold of stiffness in the allocation of Hlb-Group, whereas the LBS gradient was set based on the data of the previous study by Ortega’s team. The shoes used in Llb-Group were adjusted by changing the thickness of the carbon plate (Ortega et al., 2021).

Previous studies have demonstrated that the increasing LBS could not affect the hip angle, angular velocity, or moment of the participants. (Hoogkamer et al., 2019; Xu et al., 2022), which is inconsistent with the results of this study. According to the results of this study, it is suggested that the ROM of the hip joint would reduce under the Hlbs conditions. Moreover, the results of time-series analyses revealed that adolescent runners would show smaller initial hip angles and larger initial ankle angles in the sagittal plane under the Hlbs condition. Besides, the decrease in the ROM of the hip joint under the Hlbs condition might be induced by the underdeveloped musculoskeletal muscles of adolescents as well as their immature motor control system. The potential mechanism might be that the decreased ROM of the hip joint when wearing shoes with a carbon plate of Hlbs could compensate for the change in the angle of the knee and ankle joints. The neural adjustment ability of the teenagers’ body which could make a timely adjustment before landing is weaker than that of adults. Therefore, in this study, adolescent runners showed smaller initial hip angles and larger initial ankle angles in the sagittal plane. Many previous studies have discovered a similar phenomenon in which the increasing LBS stiffened the MTP joint, limited dorsiflexion, and slowed the angular velocity of dorsiflexion (Willwacher et al., 2013; Oh and Park, 2017; Hoogkamer et al., 2019; Xiang et al., 2022), being consistent with the findings in this study, which claimed that shoes with carbon plates of Hlbs could reduce the ROM of the MTP joint.

As mentioned above, a previous study by Roy and Stefanyshyn (2006) reported that the peak ankle moment would increase with a larger LBS during running on a treadmill with an inclination of 1%. However, some other previous studies found that there would be neither difference in the peak ankle moment or average ankle moment (Wannop et al., 2017; Beck et al., 2020). In addition, a study by Willwacher et al. (2014) published in 2014 demonstrated that running on shoes with moderate stiffness could have a lower mean ankle moment than running on control shoes (Llbs). The result of this study presented a decrease in the peak moment of the ankle joint under the Hlbs condition. Since the results of the time-series analyses revealed an increase in muscle force of the tibialis anterior muscle during the propulsive phase under the Hlbs condition and an unchanged force of plantar flexors, the reduced ankle moment was likely the consequence of the tibialis anterior forces increase. It also means that there was a greater co-contraction around the ankle, which is possibly induced by the necessity to transmit a higher amount of force generated by the increased stiffness. The cushioning effect of the shoes is mainly presented in the runner’s heel, since when the overall LBS of the shoes increased, the cushioning effect on the heel faded, and then the impact on the lower limb increased. The lower limb of an adolescent runner has an immature musculoskeletal ability for his or her self-protection. Therefore, adolescent runners tend to go through the braking phase faster and then enter the propulsive phase. In this way, under the Hlbs condition, the timing of the moment production in the hip, knee, and ankle occurred earlier, whereas that in the MTP joint had no changes. The reason might be that the running skill used was a rearfoot grounding which had little effect on the MTP during the breaking phase. This study also found that the impulse of vastus medialis, vastus lateralis, flex dig brevis, and flex hall long increased in the stance phase under the Hlbs condition. It was consistent with the hypothesis of the study that sufficient moment could not be generated to overcome the biomechanical disadvantages induced by the Hlbs carbon plate, making the contact time and impulse increase.

To sum up, it could conclude that when adolescents are running on shoes with Hlbs carbon plates, they were more likely to adopt biomechanical strategies to reduce ankle moment and contact time fhe compensation for ROM loss in their hip joints. Otherwise, the runner’s ankle plantarflexion muscle must generate more force during the stance phases to compensate for the reduced ankle moment under the Hlbs condition. Simultaneously, as the results that running on shoes with Hlbs carbon plates could increase muscle impulse by increasing the contact time, and the immature of the lower limb musculoskeletal development in adolescents, the plantarflexion muscle strength and impulse would be generated continuously when running, bringing their musculoskeletal system higher to load and increasing the risk of muscle strain and achillodynia (Best, 1993; Sancho et al., 2019). It is indicated that running shoes with Llb carbon plates would be more appropriate for young runners.

There were some limitations in this study. First, the participants of this study were all male adolescent runners. Some previous studies have demonstrated that female runners might have different biomechanical characteristics such as the different pelvic structures. Second, there were only two kinds of stiffness analyzed in this study. Further studies should explore running on shoes with carbon plates in different stiffness gradients to identify the most suitable LBS for adolescent runners.

## Conclusion

In conclusion, this study investigated the effects of different LBS shoe conditions on adolescent runners’ lower limb biomechanics and muscle mechanics. Low longitudinal bending stiffness shoes are appropriate for young runners due to musculoskeletal developmental characteristics. Further research should focus on setting multiple stiffness gradients to explore the optimal range of LBS for adolescent runners.

## Data Availability

The original contributions presented in the study are included in the article/Supplementary Material, further inquiries can be directed to the corresponding authors.
